# Breastfeeding: the hidden barrier in Côte d'Ivoire's quest to eliminate mother-to-child transmission of HIV

**DOI:** 10.7448/IAS.17.1.18853

**Published:** 2014-04-14

**Authors:** Heather M Buesseler, Ahoua Kone, Julia Robinson, Albert Bakor, Kirsten Senturia

**Affiliations:** 1 Department of Global Health, School of Public Health, University of Washington, Seattle, WA, USA; 2 Health Alliance International, Seattle, WA, USA; 3 International Training and Education Center for Health (I-TECH), Seattle, WA, USA

**Keywords:** PMTCT, HIV, adherence, ARV, prophylaxis, breastfeeding, infant feeding, qualitative

## Abstract

**Introduction:**

Côte d'Ivoire has one of the worst HIV/AIDS epidemics in West Africa. This study sought to understand how HIV-positive women's life circumstances and interactions with the public health care system in Bouaké, Côte d'Ivoire, influence their self-reported ability to adhere to antiretroviral prophylaxis during pregnancy.

**Methods:**

Semistructured interviews were conducted with 24 HIV-positive women not eligible for antiretroviral therapy and five health care workers recruited from four public clinics in which prevention of mother-to-child transmission services had been integrated into routine antenatal care.

**Results:**

Self-reported adherence to prophylaxis is high, but women struggle to observe (outdated) guidelines for rapid infant weaning. Women's positive interactions with health providers, their motivation to protect their infants and the availability of free antiretrovirals seem to override most potential barriers to prophylaxis adherence.

**Conclusions:**

This study reveals the importance of considering the full continuum of prevention of mother-to-child transmission interventions, including infant feeding, instead of focussing primarily on prophylaxis for the mother and newborn.

## Introduction

In West Africa, Côte d'Ivoire is second only to Nigeria in its HIV prevalence rate (3.4%) and deaths due to AIDS (558,900 for 1990–2009) [1,2]. The gender disparity in HIV prevalence is more pronounced in Côte d'Ivoire than in any other country in West Africa [3]: 6.4% among women compared to 2.9% among men. Over 90% of new paediatric HIV infections worldwide are transmitted vertically from mother to child during pregnancy, delivery or breastfeeding. In Côte d'Ivoire, this means that without preventive interventions, approximately 14,000 children will become infected with HIV annually [4,5]. Antiretrovirals (ARVs) are the primary line of defence for the prevention of mother-to-child transmission (PMTCT) in resource-constrained settings, since elective Caesarean delivery is unaffordable, and it is often neither safe nor acceptable to refrain from breastfeeding.

Administering ARVs requires adequately trained staff and reliable drug supplies. Over the past decade, Côte d'Ivoire has experienced various political and civil conflicts that have resulted in disruptions in care and setbacks in health sector development. At the time of this study, more than 90% of health care facilities had reopened since the 2002 civil war, but health care worker vacancy rates were still well over 50% [6]. During a four-month conflict in 2010, clinics, hospitals and laboratories were forced to close again, disrupting HIV testing and AIDS treatment and resulting in widespread drug stock-outs [7].

Following the 2002 conflict, HIV/AIDS activities in public health care facilities were largely decentralized to nongovernmental organizations (NGOs). In this system, an HIV-positive pregnant woman might have to visit up to eight sites to access prenatal consultations; delivery services; HIV testing, care and treatment; and psychosocial and nutritional support – resulting in numerous opportunities for women to be lost to follow-up in what is known as the “leaky cascade” [8,9]. Health Alliance International (HAI), an NGO based in Seattle, Washington, is collaborating with the Côte d'Ivoire Ministry of Health to improve the access to and quality of HIV prevention and treatment in public health care facilities. In 2007, HAI and Côte d'Ivoire's Ministry of Health began integrating PMTCT services at 30 public antenatal care (ANC) clinics in three districts, providing comprehensive care to HIV-positive pregnant women.

At the time of the study, World Health Organization (WHO) guidelines indicated prophylaxis regimens for all HIV-positive pregnant women not already eligible for antiretroviral therapy (ART). ART was recommended for pregnant women whose CD4 count was below 200 or for women who were in clinical stage 3 and had a CD4 count below 350 (Table 1). The recommended prophylaxis regimen included daily administration of azidothymadine (AZT) beginning at 28 weeks of pregnancy, a single dose of nevirapine during labour, followed by a single dose of nevirapine for the neonate immediately following delivery and a short course of AZT [10].

**Table 1:** Treatment guidelines for HIV-positive pregnant women at the time of the study [10]

	CD4 count		
	
WHO clinical stage	<200	200–349	350+
I	ART	Prophylaxis	Prophylaxis
II	ART	ART	Prophylaxis
III	ART	ART	ART

ART=antiretroviral therapy.

Epidemiologic studies conducted in developing countries have found that pregnant women are more likely than non-pregnant women to adhere to ARVs [11–13]. Nevertheless, Côte d'Ivoire still has low levels of ARV coverage for PMTCT. One population-based study found that just 16% of HIV-exposed infants in Côte d'Ivoire received the minimum prophylactic course of maternal-infant single-dose nevirapine – the simplest ARV regimen [14]. A recent cross-sectional survey of maternal-infant single-dose nevirapine found coverage to be low among 10 randomly selected facilities providing delivery services in the country [15].

Sociocultural factors related to the delivery and uptake of ARVs among pregnant women in resource-limited settings are an important consideration when designing and delivering effective PMTCT services [16–23]. As one study concluded, “PMTCT programs may vary in effectiveness in different contexts unless they fundamentally respond to socio-cultural factors as lived out in communities they intend to serve” [24]. The purpose of the present study was to understand how HIV-positive pregnant women's life circumstances and interactions with the health care system influence their self-reported ability to adhere to ARV prophylaxis. Specific research questions included: (1) How do HIV-positive women describe their life circumstances and interactions with the health care system during their last pregnancy? (2) What aspects of HIV-positive pregnant women's interactions with the health care unit (e.g. physical environment, patient-provider interactions, staff attitudes, etc.) impact their self-reported ability to adhere to prophylaxis? And (3) what are health care workers’ perceptions about why HIV-positive pregnant women do or do not follow through with prophylaxis?

## Methods

An exploratory, qualitative study was conducted in Bouaké, Côte d'Ivoire, the capital of the Vallée du Bandama region, using focussed ethnography [25] and principles of grounded theory [26]. Interview questions were carefully designed based on literature review and the authors’ collective professional and research experience with HIV/AIDS in Africa. The University of Washington Institutional Review Board (IRB) and Côte d'Ivoire National Ethics Committee for Life Sciences reviewed and approved this research protocol. Due to delays in local IRB approval, participant observation was not possible in this study.

### Sampling

Four public health facilities were selected for the study based on the maximum variation of clients’ ethnic background and their geographic location in Bouaké. HAI and the Côte d'Ivoire Ministry of Health had been providing these sites with PMTCT-ANC integration support for two years. At each site, two groups were sampled using criterion sampling [27]: (1) HIV-positive women aged 18 to 44 with known serostatus who had a live birth within the past 18 months and who were not eligible for ART during their last pregnancy (recruited at clinic-based support groups for HIV-positive women); and (2) health care workers who provide direct prenatal, delivery and postpartum care to HIV-positive women.

### Data collection

In-person, 45-minute, semistructured interviews were conducted at a neutral, non-clinic location in August 2009 by the first author in French (four participants were interviewed in a local language with interpreter assistance). Interview participants were reimbursed for transportation; no additional incentives were provided. Participation was voluntary, and informed consent was obtained prior to all interviews.

The interviewer is an American female with an undergraduate degree in cultural anthropology, Master’s degree in public health and significant professional experience using ethnographic methods, including designing and conducting in-depth interviews. She is fluent in French, albeit as a second language. The interviewer's interest in and reasons for the study were described to participants at the time of recruitment and during informed consent.

The local language interpreter is a male Ivoirian of similar ethnic identity as those requiring translation. His first language is the same as those for whom he translated, and he is fluent in French. He is an HAI staff member, though he had no established relationship with participants prior to the interview.

An interview guide was developed for each sampling group. In the spirit of grounded theory and theoretical sampling, questions with subsequent participants were developed based on what was learned from earlier interviews to be responsive to the data and promote a deeper investigation of the concepts being discovered [26].

All but three interviews were audio-recorded, translated and transcribed. For the three unrecorded interviews, detailed ethnographic field notes were written. Due to time constraints in the field, transcripts were not returned to participants for comment or correction.

### Analysis

Qualitative data analysis software (Atlas.ti 6.1) facilitated analysis. One coder (the first author) was involved in the analysis. First, descriptive codes were assigned to each transcript to identify similarities and differences among participants with particular attributes. Next, a thematic codebook was developed based on several readings of the transcripts; open and axial coding were then used iteratively until theoretical saturation was reached [26]. All negative cases were also explored so the data were fully conceptualized. The end goal of this analysis was “thick description” [28] and “thick analyses” [29]. Participants did not provide feedback on the findings.

## Results

### Participants

Twenty-four HIV-positive women and five health care workers were interviewed. Four of the health care workers were female midwives who provide ANC and PMTCT care; one was a male doctor. The women were primarily Muslim (*n=*15), married (*n*=12) and of Malinké ethnicity (*n*=11). They averaged 32 years old (range: 18 to 45) and had completed five years of education on average. This is consistent with the demographics of Bouaké's general population. Infants from the women's most recent pregnancy averaged nine months of age (range: two to 24 months). Although most infants were HIV-negative, five were still awaiting their test results at the time of the interview, and there was still the possibility for seroconversion among those being breastfed.

### Life circumstances related to prophylaxis adherence

#### Self-reported ability to adhere

Women self-reported strong adherence to prophylaxis, including taking prescribed medications at the appropriate times and returning for refills. One woman explained her commitment to adhere to the medication regimen:

I took the medicine in the morning at eight o'clock and also in the evening at eight o'clock. I never forget, because I am ill. I always ask someone with a watch if it is eight o'clock. My day to die has not yet come. I want to live a long life to care for my children. (Age 39, unmarried, zero years of education)

Midwives confirmed that most of their patients have no difficulty adhering to prophylaxis. Women also reported taking single-dose nevirapine dispensed to them at an ANC visit, though midwives noted that some take it too close to delivery to be effective. Women and midwives additionally revealed that most newborns received single-dose nevirapine immediately after delivery.

#### Motivation to protect infant

Women are strongly motivated to adhere to prophylaxis to protect their unborn baby. As one woman explained, “I was scared the baby would become HIV-positive, so I did everything [the midwives] told me to do” (age 37, not married, eight years of education). A few women who previously lost children to HIV were determined to avoid losing another to the illness.

#### Barriers to adherence

A few women reported an occasional difficulty with the prophylaxis regimen, such as forgetting to take the medicine or that it made them tired and weak, though they did not stop taking the medications. Although women's numerous household responsibilities continued unabated during pregnancy – including caring for children, working in the fields, earning money to buy food and cooking for the household – they reported it did not impede adherence to prophylaxis. Women did not discuss opportunity costs they incurred due to clinic visits as a barrier to obtaining or maintaining their medication regimen.

Only one woman had initially refused to take prophylaxis, but she described how strong follow-up care changed her perspective.

At first they gave me the medicine, and I threw them out. I was scared to swallow the pills. [The midwife] said, “But do you want to die?” When she said this, frankly, I was scared. I said, “Oh, so I will die and leave my children.” [The midwife] gave me more medicine. I started to take it and began to regain my form. (Age 42, widowed, six years of education)

This woman explained that she now encourages other HIV-positive pregnant women to adhere.

#### Disclosure

Refusing to accept one's serostatus can be a fundamental obstacle to adherence. One midwife described,

If she doesn't accept the result, she can't take the medications. […] She doesn't accept because she knows that her friends and family will not accept that she has this disease – they will reject her. This makes it difficult for the patient to follow the medication. I think if people accept [HIV diagnosis] easily, like someone has malaria or kidney disease or diabetes, I think it will be easier to take the medications.

Midwives suggested that this fear of rejection may be why women do not accept their diagnosis or return to the clinic for treatment. They recommended creating a psychosocial support staff position at each clinic to improve posttest counselling and reduce loss to follow-up.

Disclosing one's HIV status to a partner or family member can be paradoxical: some partners extended moral support and assisted with prophylaxis adherence, but many women had not revealed their HIV status for fear of abandonment. One health care worker explained, “She doesn't tell her husband; [the women] are afraid because the economic power lies with the man.” A few women described that after disclosure, their partners rescinded all financial support.

When I told him [that I was HIV-positive], he said not to live with him anymore; that he doesn't want anything to do with me anymore if I am sick. I pleaded with him. Up until this day, I plead with him. But he still hasn't accepted it, so he doesn't take care of me anymore. (Age 32, unmarried, zero years of education)

This potential barrier is mitigated by the availability of free medications. One woman described,

I really wasn't bothered when I got the result that I was HIV-positive. But when I started to think about the fact that both me and my husband aren't working, that's when I began to cry. But the midwives consoled me and told me the medicines were free, so I felt better after that. (Age 28, married, zero years of education)

This woman's greatest fear was not her diagnosis, but the inability to afford medication.

### Health system factors related to adherence

#### Interpersonal interactions with health care personnel

Most women described positive interactions with ANC clinic personnel.

One thing is certain—they really followed me well. I cannot speak badly about [the midwife] because she gave me her phone number in case there was a problem so I could call her and tell her. Even if she wasn't at work, she said she would meet me at the clinic. (Age 29, married, six years of education)

Since many women had not disclosed their serostatus to partners or family, midwives and support groups were their only source of support and advice. Women trust the advice of the midwives, who offer posttest encouragement and assistance, and assure the women that medications exist to prevent transmission to their babies.

Really, the only thing you need to do [to give birth to a healthy baby] is to follow the advice of the midwives. Simply do what they say. Just take their medicines as they want. That's all I can say … truly, to save that baby. (Age 22, unmarried, 13 years of education)

Although most women felt the midwives were kind and welcoming, two felt the midwives were more concerned about the health of the baby as opposed to the woman herself. Nevertheless, they still attended their ANC appointments and followed through with the midwives’ guidance for PMTCT.

#### Perceived effectiveness of prophylaxis

Women strongly believe in the effectiveness of prophylaxis in preventing HIV transmission. When asked why, many echoed this woman's reasoning: “Because the day they told me I was HIV-positive, if I continued without medicine, maybe the baby would become infected. But with the medicines they gave me, he is HIV-negative. So I trusted the medicines” (age 28, married, five years of education). Others felt prophylaxis is better than chance: “It isn't normal that you would spend nine months with a baby for something you could have avoided, and then you lose your baby. That would be terrible. Therefore, it's better to try and avoid things like that” (age 22, unmarried, 13 years of education).

Women did not report using traditional methods during pregnancy to prevent HIV transmission. They described indigenous medicines used to prevent placental malaria transmission, but acknowledged these methods do not prevent HIV transmission and admitted the midwives advised against their use.

#### Drug availability

Midwives stated that drug stock-outs at the clinics are the primary reason why women fail to adhere, though stock-outs were more common in the earlier phases of the PMTCT programme. During this study, stock-outs were rare, because a strong supply system was in place.

### Infant feeding

Respondents emphasized that infant feeding – not prophylaxis adherence – is the real threat to vertical transmission. This emerged even though the original interview guide did not address this topic. When it became clear infant feeding was a significant concern, questions were subsequently added.

Women knew that the recommended guidelines at the time of the study were exclusive breastfeeding through the sixth month followed by rapid weaning. However, many reported that financial barriers and associated stigma or fear of disclosure prohibited adherence to these guidelines. One woman summarized,

Breastfeeding – that is the problem. Imagine a woman who doesn't want to tell her husband she is HIV-positive. He is going to ask her why she stops breastfeeding. […] So what do you tell him? And then when he must go buy milk, and then he will say he doesn't have money and all that. How will the infant fare? Oh! That is the problem. (Age 22, unmarried, 13 years of education)

Participants explained it is common for HIV-negative women to breastfeed for one to two years; thus, not breastfeeding or weaning at six months raises difficult questions from partners, family, friends and neighbours.

Some women said that after six months, they fed formula when they could afford it but otherwise reverted to breastfeeding. A few women, however, found ways to wean at the appropriate time.

When I found out about my [HIV] status, I began to set aside money. I saved up until the birth so that the day of the birth I could buy milk for the baby until he was five months old. Then I made his father understand that my breasts weren't producing any more milk and I couldn't breastfeed my baby. It was then he started to help me, up until today. (Age 33, cohabiting with partner, six years of education)

Midwives described that for mothers struggling financially, it often becomes a choice between a lesser of two evils – HIV transmission or malnutrition.

We are in a situation of extreme poverty, yet we emphasize to women that they must stop breastfeeding. But if they don't have anything and she stops, the baby will die of malnutrition. It isn't HIV that will kill the baby, it's malnutrition. […] We no longer tell them to stop breastfeeding because when you see [the babies’] condition, if you tell them to stop breastfeeding the infant will become malnourished. You often have to breastfeed with all the risks.

## Discussion

Women's greatest struggle in preventing vertical HIV transmission was adherence to infant-feeding guidelines. Proper infant feeding is an important element of the continuum of PMTCT interventions. WHO calls it “one of the most critical interfaces between HIV and child survival” [30]. Though nearly all women knew they should not breastfeed or wean rapidly at six months, most could not because replacement feeding was too costly, and given cultural norms to breastfeed for up to two years, early weaning risked HIV status disclosure.

For many low-resource countries grappling with HIV, implementing rapid weaning recommendations for HIV-positive women has been a challenge [31–34]. These countries encountered similar dilemmas as reported in this study – the psychosocial distress associated with a mother's choice between possible HIV infection and the morbidity and mortality associated with malnutrition. In 2010 and 2012, WHO made a series of updates to PMTCT guidelines, including infant-feeding recommendations [30,35]. It now recommends that for countries that have chosen breastfeeding and ARV interventions (as opposed to replacement feeding) as the national strategy,

Mothers known to be HIV-infected (and whose infants are HIV uninfected or of unknown HIV status) should exclusively breastfeed for the first six months of life, introducing appropriate complimentary foods thereafter, and continue breastfeeding for the first 12 months of life. Breastfeeding should then only stop once a nutritionally adequate and safe diet without breast milk can be provided. [35]

In October 2012, the Ivoirian Ministry of Health adopted these guidelines in its own revised protocol [36]. However, one cannot assume a smooth transition from policy to programming. Although update trainings have been conducted for health care workers throughout the country, Eamer asserts that WHO's new guidelines do not necessarily address the structural barriers that prevented earlier infant-feeding recommendations from being effective [37]. Without addressing these implementation gaps, changes in infant-feeding practices may be limited. Additional research is needed to more fully understand these challenges in an effort to achieve WHO's call to eliminate HIV in children worldwide by 2015.

Women in this study reported high levels of adherence to prophylaxis during pregnancy and to the infant dose of prophylaxis immediately following delivery. This is noteworthy, considering that a recent epidemiologic study in Côte d'Ivoire [14] reported very low adherence. Our research reveals that women receiving care from health centres with integrated ANC and PMTCT services report they are able to adhere to prophylaxis regimens. While power dynamics, stigma and poor interpersonal skills between health care workers and patients are common themes in the literature [21,38,39], women here reported good treatment, guidance and moral support from the midwives. This answers WHO's call for quality services and integration of PMTCT into other maternal and newborn services in the PMTCT Strategic Vision 2010–2015 [40], and it fulfils the Ivoirian Ministry of Health's mandate.

Both women and midwives in this study stressed acceptance of serostatus as an important first step in pursuing a course of treatment. Reducing loss to follow-up after the delivery of HIV-positive test results, therefore, is critical to ensuring prophylaxis uptake. Midwives suggested adding an on-site psychosocial support position at each clinic to provide more in-depth posttest counselling and follow-up.

High self-reported adherence in this study is consistent with population-based studies conducted in other developing country settings, where pregnant women are more likely than non-pregnant women to adhere to ARVs [11–13]. Despite a lack of social support for their HIV diagnosis and unabated household responsibilities during their pregnancy, women in our study remained committed to their infants’ health. The availability of free ARVs is critical, given women's significant financial struggles and some experiences of partner rejection upon HIV status disclosure. Without free medications, significantly more women are likely to struggle to adhere to prophylaxis where relatively few barriers were otherwise reported.

Fortunately, drug stock-outs – one of the only prophylaxis adherence barriers revealed in this study – were reportedly less frequent during this research period than when integrated ANC and PMTCT services were initially introduced. However, recurrent political instability will continue to impede reductions in mother-to-child transmission – drugs were in short supply during the 2010 conflict, and stock-outs doubled the risk of HIV treatment discontinuation or death during the civil war [41].

## Limitations

Selection bias may affect this study. Due to recruitment methods and time limitations, women outside of support groups, not receiving public ANC services, or lost to follow-up were not included in the study. Such women may have systematically different experiences at the ANC clinics that may have caused them to drop out of the system or to not adhere to prophylaxis. Since women were recruited through ANC clinic support groups, their responses may be skewed toward positive experiences with the health care system and a stronger biomedical orientation. Potential selection bias in this study does not diminish the importance of the findings – for women who maintain a relationship with the health care system, it appears to serve them well and facilitate antenatal adherence to prophylaxis. Future research should focus on identifying non-adherent women or women lost to follow-up to determine how their experiences differ from those of women in this study.

Social desirability bias may have influenced some women's responses to questions about adherence, though care was taken to design questions that were not leading, and interviews were conducted away from the health clinic. Nevertheless, it is possible women perceived the interviewer to be closely linked to clinic staff, thereby limiting their inclination to be critical of the care they received.

Regarding the validity of self-reports, this study did not have the capacity to objectively examine women's reported prophylaxis adherence. However, midwives in this study corroborated women's self-reports; furthermore, a study conducted in Zambia found 75% of women accurately reported ARV adherence during pregnancy [42].

Another limitation of the study is the small number of health care workers interviewed. Had time allowed, interviewing additional health care workers may have revealed a greater range of responses and contributed to a deeper understanding of the complex and contingent nature of provider-patient relationships within the clinical context.

## Conclusions

Most potential barriers to adherence were overcome by women's motivation to protect their infant's health coupled with the availability of free ARVs. Strong ties to the public health care system and high-quality ANC and PMTCT services also facilitate pregnant women's adherence to prophylaxis. However, women in the study struggled to adhere to outdated infant-feeding recommendations. Although the Côte d'Ivoire's PMTCT protocol has been updated and health workers trained, in order to effect changes in infant-feeding practices, research is needed to understand the structural challenges in implementing both former and current infant-feeding guidelines. Midwives asserted that adding on-site clinic staff – especially for infant-feeding counselling, posttest counselling and tracking women lost to follow-up – is also necessary to further reduce PMTCT. If political instability reoccurs, reductions in vertical transmission may slow or stagnate due to service interruptions and drug stock-outs. This study reveals the importance of considering the full continuum of PMTCT interventions, including infant feeding, instead of focussing primarily on prophylaxis for the mother and newborn.
